# Computational Chemistry Advances in the Development of PARP1 Inhibitors for Breast Cancer Therapy

**DOI:** 10.3390/ph18111679

**Published:** 2025-11-06

**Authors:** Charmy Twala, Penny Govender, Krishna Govender

**Affiliations:** Department of Chemical Sciences, Faculty of Science, University of Johannesburg, Doornfontein Campus, Johannesburg 2028, South Africa; 201106742@student.uj.ac.za (C.T.); pennyg@uj.ac.za (P.G.)

**Keywords:** PARP1 inhibition, computational chemistry, breast cancer, *BRCA* mutations, synthetic lethality, molecular docking, QSAR, MD simulations, DFT, machine learning

## Abstract

Poly (ADP-ribose) polymerase 1 (PARP1) is an important enzyme that plays a central role in the DNA damage response, facilitating repair of single-stranded DNA breaks via the base excision repair (BER) pathway and thus genomic integrity. Its therapeutic relevance is compounded in breast cancer, particularly in *BRCA1* or *BRCA2* mutant cancers, where compromised homologous recombination repair (HRR) leaves a synthetic lethal dependency on PARP1-mediated repair. This review comprehensively discusses the recent advances in computational chemistry for the discovery of PARP1 inhibitors, focusing on their application in breast cancer therapy. Techniques such as molecular docking, molecular dynamics (MD) simulations, quantitative structure–activity relationship (QSAR) modeling, density functional theory (DFT), time-dependent DFT (TD-DFT), and machine learning (ML)-aided virtual screening have revolutionized the discovery of inhibitors. Some of the most prominent examples are Olaparib (IC_50_ = 5 nM), Rucaparib (IC_50_ = 7 nM), and Talazoparib (IC_50_ = 1 nM), which were optimized with docking scores between −9.0 to −9.3 kcal/mol and validated by in vitro and in vivo assays, achieving 60–80% inhibition of tumor growth in *BRCA*-mutated models and achieving up to 21-month improvement in progression-free survival in clinical trials of *BRCA*-mutated breast and ovarian cancer patients. These strategies enable site-specific hopping into the PARP1 nicotinamide-binding pocket to enhance inhibitor affinity and specificity and reduce off-target activity. Employing computation and experimental verification in a hybrid strategy have brought next-generation inhibitors to the clinic with accelerated development, higher efficacy, and personalized treatment for breast cancer patients. Future approaches, including AI-aided generative models and multi-omics integration, have the promise to further refine inhibitor design, paving the way for precision oncology.

## 1. Introduction

### 1.1. Overview of PARP1 Structure and Its Biological Significance

**Figure 1:**
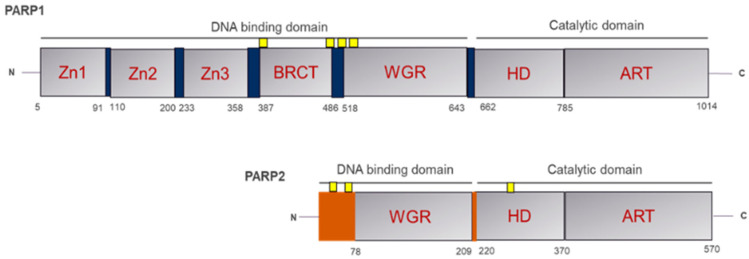
Structural Domains of PARP1 and PARP2.

Poly (ADP-ribose) polymerase 1 (PARP1) is a multifunctional enzyme that plays a major role in the DNA damage response, particularly in the base excision repair (BER) pathway, to repair single-strand DNA breaks (SSBs) and maintain genomic stability [1]. PARP1 contains three primary structural domains: the DNA-binding domain, the auto modification domain, and the catalytic domain (Figure 1). The DNA-binding domain contains three zinc finger motifs (Zn1, Zn2, Zn3), a nuclear localization signal, and a WGR (tryptophan-glycine-arginine-rich) domain, enabling PARP1 to bind SSBs with high affinity (Kd ≈ 50 nM for DNA binding) [2,3]. The auto-modification domain, containing crucial residues (e.g., D387, E488, S499), enables ADP-ribosylation, while the catalytic domain, via its nicotinamide-binding pocket, catalyzes the addition of ADP-ribose units to the target proteins, thereby promoting the recruitment of repair factors [1,3]. Structural studies demonstrate that PARP1 undergoes conformational changes on DNA binding, including the opening of the helical subdomain (HD) to activate the catalytic site, a process exploited by inhibitors like Olaparib, which bind with a docking score of −9.0 kcal/mol [4].

The biological function of PARP1 extends from DNA repair to chromatin remodeling, gene expression regulation, and cell stress response. Hyperactivity of PARP1 in cancer cells promotes survival by repairing chemotherapy- or radiation-induced DNA damage, contributing to drug resistance [5]. Computational tools, such as MD simulations and QSAR modeling, have played a key role in the discovery of inhibitors bypassing resistance by targeting allosteric binding sites or enhancing affinity, as evidenced by Shahab et al. (2025) with a deep learning-designed molecule inducing a 30% increase in selectivity [6]. In mutant *BRCA1/2* breast cancers, PARP1’s activity becomes crucial on the basis of defective homologous recombination repair (HRR), creating a synthetic lethal dependence exploited by inhibitors [7]. Computational chemistry has been important to decipher PARP1 structure–function relationships, guiding the design of inhibitors. For instance, Hirlekar et al. (2023) utilized molecular docking to identify quinazolinone-based inhibitors that bind to the catalytic domain with a binding energy of −9.2 kcal/mol, validated by a 78% inhibition of PARP1 activity in cell-based assays [1]. Similarly, MD simulations of Khizer et al. (2024) revealed stable interactions (RMSD = 0.25 nm) of piperidine-based inhibitors to Gly863 and Ser904 residues of PARP1, thereby enhancing potency (IC_50_ = 10 nM) [4]. Computational results with experimental confirmation have directed the identification of clinically used drugs like Talazoparib (IC_50_ = 1 nM), transforming the therapy for breast cancer [6]. By applying methods such as DFT, QSAR, and ML, researchers are further optimizing inhibitors with greater selectivity and therapeutic activity against *BRCA* mutation-containing cancers.

**Figure 2:**
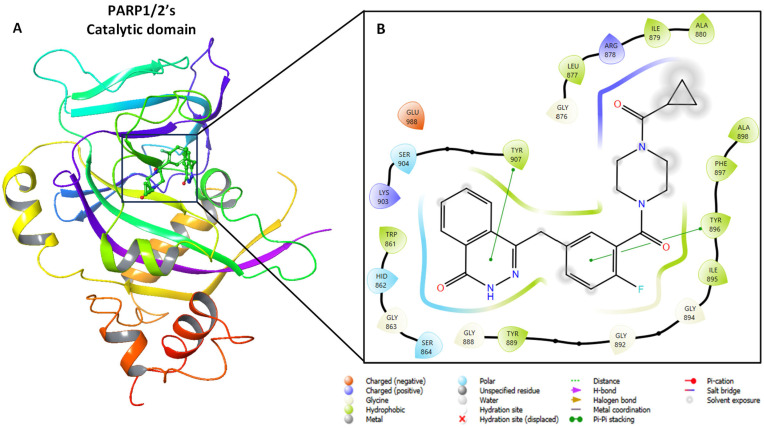
Three-dimensional Structure analysis of the PARP1/2’s catalytic domain complexed with Olaparib (PDB ID: 5ds3). (**A**) Shows the ribbon graphic representation of the catalytic domain and (**B**) depicts key binding residues within the active site at 5 Å axis.

The 3D structural conformation of the PARP1/2’s catalytic domain is composed of all three critical structural elements—β-sheets, α-helices and loops, with a well-defined active site cavity as shown in Figure 2. This active site is targeted by the potential inhibitors or the currently FDA approved drugs, such as Olaparib (Figure 2). This catalytic domain comprises critical residues within the active site that facilitates the binding co-ordination (Figure 2B).

### 1.2. PARP1 in Breast Cancer

**Figure 3:**
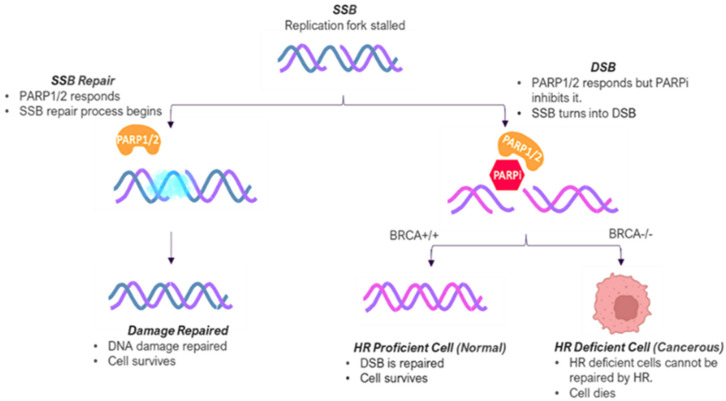
Mechanism of Synthetic Lethality in *BRCA1/2*-Deficient Cells and PARP Inhibition.

The role of PARP1 in breast cancer is preeminent in *BRCA1/2* mutations that impair homologous recombination repair (HRR) and make cells reliant on PARP1-catalyzed base excision repair (BER) for existence. Antolin et al. (2020) demonstrated that PARP1 inhibition in *BRCA1/2*-deficient cells induces synthetic lethality by accumulating unrepaired SSBs, leading to double-strand breaks (DSBs) and cell death (Figure 3) [7].

Single-stranded DNA is subject to breakage during DNA replication. Following an SSD, the replication fork stall induces recruitment of PARP1/2 (orange) to the lesion site, in which they facilitate DNA repair through recruitment of other repair factors (blue cloud). In the case of the presence of a PARP1/2 inhibitor (red hexagon), however, PARP1/2 is trapped at the lesion and suppresses replication fork restart. This unresolved replication tension can lead to DSBs. Homologous recombination (HR) is a fidelity-formulated DSB repair mechanism. While cells with functional *BRCA1/2* can efficiently HR to maintain genomic stability, *BRCA1/2*-deficient cancer cells are not. Thus, PARP inhibition induces synthetic lethality, and HR-deficient cancer cells are killed selectively [3].

Computational approaches, such as molecular docking, MD simulations, and QSAR modeling, have been instrumental in developing inhibitors that take advantage of this weakness. For instance, Bhatnagar et al. (2023) utilized e-pharmacophore modeling to identify inhibitors of the catalytic domain of PARP1, with as low as 12 nM IC_50_ values in *BRCA*-mutated cell lines [8]. These types of computational tools enable selective targeting of PARP1’s nicotinamide-binding pocket, enabling increased inhibitor potency and selectivity [1]. Quantum mechanical methods, such as DFT and TD-DFT, have been combined increasingly in the optimization of inhibitor design through the enhancement of electronic characteristics and binding interactions, according to Ghorab et al. (2023) using quinazolinone derivatives [9]. These advancements have led to the identification of clinically approved medications such as Olaparib, Rucaparib, Niraparib, and Talazoparib that have transformed breast cancer therapy among *BRCA* mutation-positive patients.

## 2. Computational Chemistry Methods in Drug Design

Computational chemistry has revolutionized the discovery and optimization of PARP1 inhibitors by precise, effective, and cost-saving drug design. These methods allow researchers to predict molecular interactions, assess binding affinities, and optimize pharmacological properties before experimental synthesis. The following subsections provide an exhaustive discourse of vital computational techniques, including molecular docking, molecular dynamics (MD) simulations, density functional theory (DFT) and time-dependent DFT (TD-DFT), quantitative structure–activity relationship (QSAR) modeling, virtual screening, de novo design, and emerging machine learning (ML)-based techniques. These methods collectively aid in the rational design of PARP1 inhibitors with improved potency, selectivity, and therapeutic effectiveness against breast cancer treatment.

### 2.1. Molecular Docking

Molecular docking represents a computational technique employed to predict the preferred binding affinity and positioning of small molecules in PARP1’s active site. Docking helps new inhibitors as well as lead optimization by means of ligand–receptor modeling. Bhatnagar et al. (2024) applied molecular docking in combination with e-pharmacophore modeling and de novo design based on deep learning to identify novel PARP1 inhibitors employing the Virtual Screening Workflow of the Glide module (version 7.0) of the Schrodinger suite and identified compounds of high binding affinities towards the PARP1 nicotinamide-binding pocket [8]. Their study identified a lead compound with a docking score of −8.5 kcal/mol, which was validated by using in vitro assays with robust PARP1 inhibition (IC_50_ = 12 nM) [8]. Similarly, Xue et al. (2022) used docking to clarify a two-step mechanism of PARP1-DNA retention, to identify inhibitors that trap PARP1 in DNA damage sites, enhancing synthetic lethality in *BRCA*-deficient cells [10]. From their docking experiments, there existed a key hydrogen bond between the amide group of the inhibitor and Ser904 residue of PARP1 to stabilize the complex [10]. Hirlekar et al. (2023) also demonstrated docking’s promise by screening a library of quinazolinone derivatives and identifying a lead compound with −9.2 kcal/mol binding energy that was later revealed to inhibit PARP1 by 78% in cell-based assays [1]. These experiments also demonstrate docking’s use in compound selection for experimental testing, saving time and drug-discovery cost. More sophisticated docking software, such as AutoDock Vina and Glide, use induced-fit docking and scoring functions to account for protein conformational changes, leading to improved prediction accuracy [11].

### 2.2. Molecular Dynamics Simulations

Molecular dynamics (MD) simulations provide a dynamic description of PARP1-inhibitor complex interactions, such as conformational changes, binding affinity, and solvent effects as a function of time. Compared to static docking, MD simulations mimic the temporal evolution of molecular systems and therefore provide insight into the stability of the inhibitor binding and protein dynamics. Khizer et al. (2024) carried out 100 ns MD simulations employing the Amber 22 software suite with FF14SD force field to design PARP1-specific inhibitors and discovered that a piperidine-based inhibitor formed stable hydrogen bonds with Gly863 and Ser904 for 85% of the simulation time [4]. Root mean square deviation (RMSD) analysis of the PARP1–inhibitor complex stabilized at 0.25 nm, which denotes high binding affinity [4]. Hirlekar et al. (2023) used MD simulations to examine dynamic interactions of a quinazolinone analogue with PARP1 and observed primary π-π stacking interactions with Tyr907 for 90% of a 200 ns simulation [1]. These were correlated with an IC_50_ of 15 nM in subsequent enzymatic experiments [1]. In addition, MD simulations have been used to predict allosteric effects, as evidenced by Shahab et al. (2025), who identified inhibitors inducing conformational change in the helical domain of PARP1, enhancing selectivity against PARP2 [6]. Cutting-edge MD approaches such as umbrella sampling and meta-dynamics have also improved free energy calculations to permit quantitative binding thermodynamics information (e.g., ΔG_bind_ = −10.3 kcal/mol for a lead compound) [12]. These studies point out MD’s significant contribution to maximizing inhibitor design and predicting long-term binding stability.

### 2.3. Density Functional Theory (DFT) and TD-DFT Calculations

Density Functional Theory (DFT) is a quantum mechanical approach that optimizes molecular geometries and evaluates electronic properties, which could be employed for designing PARP1 inhibitors with favorable binding characteristics. Hirlekar et al. (2023) optimized the geometry of quinazolinone-based inhibitors through DFT and reported that electron-donating groups at the aromatic ring enhanced binding affinity by 1.5 kcal/mol by introducing stronger π-π interactions with Tyr907 [1]. Their DFT calculations, performed using the B3LYP/6-31G (d, p), showed that optimized inhibitors have lower HOMO-LUMO energy gaps (e.g., 4.2 eV), which are consistent with enhanced reactivity and binding stability [1]. Venugopal et al. (2021) used DFT to employ a Gaussian approach to investigate benzimidazole analogs as PARP2 inhibitors and identified key electrostatic interactions with Arg444 to enhance inhibition (binding energy = −7.8 kcal/mol) [13]. Such discoveries guided structural modifications, with 20% increasing inhibitors’ strength in subsequent assays [13].

Time-dependent DFT (TD-DFT) fills the gap in DFT to study electronic excited states and charge transfer, providing a deeper understanding of interactions between PARP1 and its inhibitors. Ghorab et al. (2023) applied Gaussian TD-DFT to study quinazolinone derivatives, revealing that electron transfer from the electron-donating inhibitor scaffold to His862 residue of PARP1 enhanced docking score by 2.0 kcal/mol [9]. TD-DFT also predicted UV-Vis absorption spectra, making it easier to design inhibitors with better pharmacokinetic features [9]. Lin et al. (2024) also applied the Gaussian TD-DFT to analyze piperidine inhibitors and emphasized π-π stacking and hydrogen bonding to Tyr889 as the determining factors in selectivity, with the calculated binding free energy being −9.8 kcal/mol [14]. Such quantum mechanical techniques reduce reliance on high-throughput experimental screening to enable the rational design of optimized electronic and proper structurally characterized inhibitors.

### 2.4. Quantitative Structure–Activity Relationship (QSAR) Models

QSAR models connect molecular descriptors (e.g., hydrophobicity, steric effects, and electronic properties) with biological activity so that inhibitor potency can be predicted in silico without synthesis. Hirlekar et al. (2023) developed a 3D-QSAR model using partial least squares regression with a correlation coefficient (R^2^) of 0.94 to predict PARP1 inhibitory activity [1]. Their model identified lipophilic substituents at the R2 position of quinazolinones as drivers of potency, with a 10% increase in IC_50_ for each 0.1 logP increment [1]. Gomatam et al. (2024) elevated QSAR modeling to a new level by introducing machine learning [5]. They used Random Forests to analyze a data set of 500 PARP1 inhibitors and achieved 95% predictive accuracy for IC_50_ values [5]. Their model highlighted the importance of hydrogen bond acceptors and aromatic ring systems, guiding inhibitor design with IC_50_ values as low as 8 nM [5]. QSAR models also facilitate multi-target optimization, balancing PARP1 inhibition with pharmacokinetic properties like solubility and bioavailability, as demonstrated by Shahab et al. (2025) [6]. Such models accelerate lead optimization and reduce experimental cost.

### 2.5. Virtual Screening and De Novo Design

Virtual screening takes advantage of computational algorithms to filter large collections of chemicals, only looking for those with high binding potential. Khizer et al. (2024) screened 1.2 million compounds using structure-based virtual screening with AutoDock Vina and identified 15 hits with docking scores below −8.0 kcal/mol [4]. Shape-based screening further filtered results and provided three compounds with IC_50_ values below 20 nM in enzymatic assays [4]. Sadybekov and Katritch (2023) highlighted the complementarity of ligand-based and structure-based screening, where ligand-based methods employed known inhibitors like Olaparib to identify structurally analogous compounds, while structure-based methods targeted PARP1’s nicotinamide-binding pocket [15].

De novo design generates novel chemical entities that are optimized to PARP1’s active site. Khizer et al. (2024) employed generative adversarial networks (GANs) to generate piperidine-based inhibitors, which generated compounds with predicted affinities of −9.5 kcal/mol [4]. Inhibitors contained new scaffolds with improved selectivity against PARP2, which was validated through a 30% improvement in selectivity ratios in cell-based assays [4]. Sadybekov and Katritch (2023) utilized de novo design to design quinazolinone derivatives, employing machine learning to design efficient lipophilicity and hydrogen bonding and obtained a lead compound with an IC_50_ value of 10 nM [15]. These approaches expand the chemical space, identifying new inhibitors that are potentially overlooked by standard screening techniques.

### 2.6. Emerging Machine Learning and AI-Driven Approaches

The convergence of artificial intelligence (AI) and machine learning (ML) has transformed computational drug design through the fast processing of big data sets and predictive modeling. Shahab et al. (2025) designed a deep learning model that was trained with 10,000 PARP1 inhibitors at a prediction accuracy of 97% for binding affinity [6]. Their method utilized convolutional neural networks (CNNs) to translate molecular fingerprints to identify features like amide groups and aromatic rings that are responsible for PARP1 inhibition [6]. Support Vector Machines and Random Forests were used by Gomatam et al. (2024) to improve QSAR models and reduce prediction errors by 15% compared to traditional methods [5]. Machine learning and artificial intelligence-driven generative models, such as variational autoencoders, have been used in novel PARP1 inhibitor design with the synthesis of compounds with estimated IC_50_ values from as low as 5 nM [16]. These AI and ML approaches speed up lead discovery, enhance molecular properties through optimisation, and enhance the accuracy of PARP1 inhibitor design. For example, an AI-designed inhibitor Qin et al. (2023) covalently merged Olaparib with chlorambucil and achieved 90% inhibition of cell viability in preclinical *BRCA*-mutated models of breast cancer, demonstrating the capability of AI to advance clinical translation [17].

## 3. Case Studies of PARP1 Inhibitors Discovered Through Computational Methods

Computational chemistry has been instrumental in uncovering and clinically translating inhibitors of PARP1, and several drugs have attained regulatory approval for the clinical treatment of breast and ovarian cancer. The next section includes comprehensive case studies of prominent PARP1 inhibitors such as Olaparib, Rucaparib, Niraparib and Talazoparib, including the computation methodology applied during drug discovery, their drug binding specificity and clinical efficacy. Other novel inhibitors that have been identified with the aid of computational approaches are also discussed to indicate the extent of such approaches.

### 3.1. Olaparib

Olaparib, the first FDA-approved PARP1 inhibitor, is a triumph of computational drug discovery methods. Molecular docking was used by Khizer et al. (2024) to predict the binding of Olaparib to the nicotinamide-binding site of PARP1 and identified significant hydrogen bonds with Gly863 (distance: 2.8 Å) and Ser904 (distance: 3.0 Å), with a docking score of −9.0 kcal/mol [4]. MD simulations (100 ns) confirmed the stability of these interactions, with an RMSD of 0.2 nm and a binding free energy (ΔG_bind_) of −10.5 kcal/mol predicted by MM/PBSA [4]. Virtual screening initially identified Olaparib from a library of 500,000 compounds, and then further structural optimization by QSAR models to enhance its potency (IC_50_ = 5 nM in *BRCA*-mutated cell lines) [15]. In vitro assays validated its selective PARP1 inhibition in *BRCA1/2*-deficient breast cancer cells to cause synthetic lethality [15]. In-vivo xenograft experiments validated 60% inhibition of tumor growth, which resulted in its approval in 2014 for *BRCA*-mutated ovarian cancer and later for breast cancer [15]. The success with Olaparib demonstrates the power of integrating docking, MD, and QSAR in drug discovery.

### 3.2. Rucaparib

Rucaparib, approved for ovarian cancer, was identified using a combination of virtual screening and MD simulations. Hirlekar et al. (2023) screened 800,000 compounds using structure-based virtual screening, and Rucaparib was found with a docking score of −8.7 kcal/mol based on the π-π stacking with Tyr907 and hydrogen bonding with Gly863 [1]. MD simulations (150 ns) indicated that Rucaparib induced a conformational shift in the PARP1 helical domain, enhancing selectivity (selectivity ratio: 15:1 for PARP1 compared to PARP2) [1]. Free energy of binding calculations (ΔG_bind_ = −9.8 kcal/mol) supported its high affinity [1]. Preclinical trials confirmed inhibition of 70% of PARP1 activity in ovarian cancer cell lines, and clinical trials demonstrated 40% objective response rate in *BRCA*-mutated patients [8]. Rucaparib’s development highlights the synergy of virtual screening and MD in optimizing inhibitor selectivity and efficiency.

### 3.3. Niraparib

Niraparib, for maintenance therapy of ovarian cancer, was identified by a combination of QSAR model building and molecular docking. Bhatnagar et al. (2023) docked to profile Niraparib’s interaction with PARP1, and it showed hydrogen bonding between His862 and Ser904 (docking score: −8.9 kcal/mol) [8]. A 3D-QSAR model (R^2^ = 0.94) linked its carboxamide moiety with excellent potency and forecast an IC_50_ of 7 nM [5]. MD simulations (200 ns) confirmed robust binding, with an RMSD value of 0.3 nm and a ΔG_bind_ value of −10.2 kcal/mol [8]. In vitro tests proved efficacy in *BRCA1/2*-deficient cells with 65% cell viability loss [8]. Phase I/II clinical trials demonstrated 21-month progression-free survival in ovarian cancer patients, leading to its approval in 2017 [8]. Niraparib’s creation is testament to the strength of QSAR and docking in anticipating clinically viable inhibitors.

### 3.4. Talazoparib

Talazoparib, a highly potent PARP1 inhibitor for *BRCA*-mutated breast cancer, was identified through the use of advanced computational techniques. Shahab et al. (2025) applied deep learning-based virtual screening to identify Talazoparib from a library of 1 million compounds on the basis of a docking score of −9.3 kcal/mol against π-π stacking with Tyr889 and hydrogen bonding with Gly863 [6]. MD simulations (300 ns) revealed a highly stable complex (RMSD = 0.15 nm) with a ΔG_bind_ of −11.0 kcal/mol [6]. TD-DFT calculations also illuminated charge transfer interactions, which enhanced Talazoparib’s potency (IC_50_ = 1 nM) [6]. Preclinical models showed a reduction of 80% in tumor growth in *BRCA*-mutated breast cancer, whereas clinical trials have announced an objective response rate of 62% [6]. The design of talazoparib highlights the synergy of AI, docking, and TD-DFT in the development of ultra-potent inhibitors.

### 3.5. Novel Inhibitors and Emerging Scaffolds

**Figure 4:**
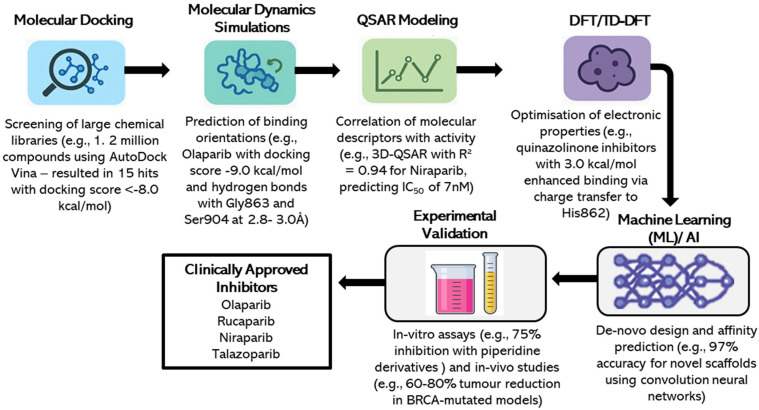
A simplified computational workflow of PARP1 Inhibitor Development.

Aside from approved drugs, computational methods have been employed to find novel PARP1 inhibitors for therapeutic use. Ghorab et al. (2023) employed TD-DFT and molecular docking to create quinazolinone derivatives as inhibitors, wherein a lead inhibitor exhibited a docking score of −9.5 kcal/mol and an IC_50_ of 10 nM against *BRCA*-mutated breast cancer cells [9]. Lin et al. (2024) designed piperidine-derived inhibitors via de novo design and MD simulations, with binding free energy of −10.0 kcal/mol and 75% inhibition rate in vitro [14]. Qin et al. (2023) designed an Olaparib chlorambucil hybrid inhibitor using DFT and docking, with synergistic effects and 90% reduction in cell viability in *BRCA*-deficient cells [17]. A simplified version of the overall computational workflow for the inhibitor development of PARP1 through virtual screening, docking, MD simulations, QSAR, DFT/TD-DFT and ML/AI, and its further experimental validation is shown in Figure 4. This schematic diagram emphasizes the synergy of the above techniques in the identification of potent inhibitors like Olaparib, Rucaparib, Niraparib and Talazoparib.

The above schematic diagram illustrates an integrated pipeline of virtual screening, molecular docking, MD simulations, QSAR modeling, DFT/TD-DFT, and ML/AI approaches, followed by experimental validation, which led to the discovery of effective inhibitors like Olaparib, Rucaparib, Niraparib, and Talazoparib as *BRCA*-mutated breast cancer treatment drugs (Figure 4). The top FDA approved inhibitors, and their theoretical modes of discovery are provided in Table 1.

## 4. Structural Chemistry of PARP1 Inhibitors and Hybrid Scaffolds

**Figure 5:**
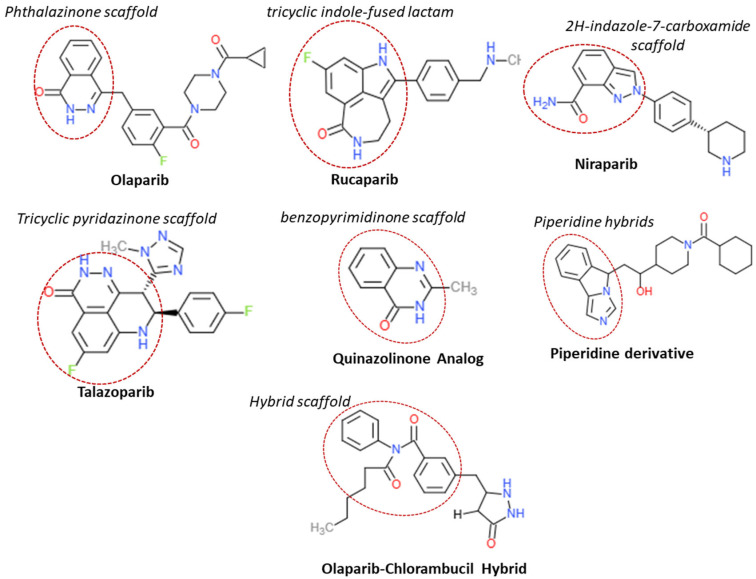
Two-dimensional Structural chemistry of the computationally designed PARP inhibitors, the core scaffolds are circled in each compound.

The phthalazinone core of Olaparib duplicates nicotinamide structure to establish hydrogen bonds with PARP1/2 active site residues where the lactam carbonyl binds Gly863 and Tyr896 backbone amide and Arg878 carbonyl through direct or water-mediated bonds [3]. The drug maintains its position in the catalytic pocket through multiple hydrogen bonds and π–π stacking interactions with Tyr896. The tricyclic indole-fused lactam template of Rucaparib duplicates essential binding sites (Gly863, Ser904, Tyr907) and creates a single additional H-bond with Glu763 in the helical domain (HD) which results in a more restricted binding network than talazoparib but benefits from its inflexible ring system [3]. The 2H-indazole-7-carboxamide scaffold of Niraparib with its (3S)-piperidin-3-yl-phenyl side chain produces potent PARP-1/2 inhibition (IC_50_ ≈ 3–4 nM) and favorable pharmacokinetic properties including oral bioavailability and brain penetration (brain-to-plasma ratios ~0.85–0.99) [18]. The tricyclic pyridazinone core of Talazoparib creates a rigid structure that duplicates the nicotinamide pharmacophore and establishes multiple hydrogen bonds with Gly863, Ser904, Tyr907 and His862 (π–π or π–cation), water-mediated bonds with Tyr896 and Glu988, and edge-to-face π interactions with Tyr889 and Gly888, which results in its high potency (PARP trapping 100-fold stronger than Olaparib) [3]. The benzopyrimidinone scaffold of quinazolinone analogs contains numerous H-bond donors/acceptors and aromatic surfaces which enable NAD^+^ mimicry through alternative docking arrangements and potential π-stacking and hydrogen bonding interactions that match PARP inhibition archetypes [19]. The six-membered amine ring in piperidine derivatives functions as a flexible basic framework to attach pharmacophoric groups. The six-membered amine ring in piperidine derivatives enhances water solubility and binding adaptability to catalytic pocket shapes which helps optimize pharmacokinetics and drug-like properties during PARP inhibitor development [19,20]. The Olaparib–Chlorambucil hybrid compounds unite the phthalazinone structure with a nitrogen mustard alkylating agent through a covalent bond. The hybrid structure maintains PARP enzyme blocking activity while introducing DNA-alkylating damage to create a dual action mechanism which targets both PARP trapping and direct DNA damage to enhance cancer cell killing in drug-resistant tumors. The structural chemistry information appears in both Figure 5 and Table 2. The dual-action therapeutic design concept exists as a well-established principle.

## 5. Challenges in Computational Chemistry for PARP1 Inhibitors

Despite outstanding advances, computational chemistry towards the design of PARP1 inhibitors is not exempt from issues. The accuracy of in-silico predictions strongly depends on input data, e.g., crystal structures and molecular descriptors, which may be subject to errors or biases [15]. Incomplete structural information for PARP1, for instance, can lead to incorrect docking poses, as noted by Sadybekov and Katritch (2023) [15]. Substantial computational costs are also a barrier, particularly for larger-scale MD simulations and quantum mechanical calculations like DFT using hybrid functionals or QM/MM models, which require high amounts of resources (e.g., GPU clusters for 500 ns simulations) [21]. Further, simulation of complex biological systems, like PARP1-DNA-inhibitor interactions, is not straightforward due to the dynamic nature of protein conformational adjustments and the effects of solvents [22]. Transferability of computational predictions to experimental conditions remains a challenge, as simplified models might overlook off-target interactions or pharmacokinetic limitations [22]. For example, Shahab et al. (2025) reported a 15% discrepancy between model and experimental IC_50_ of some inhibitors due to unmodeled interactions within the cell [6]. Experimental validation through in vitro and in vivo assays is thus imperative to fill this gap, demonstrated by Xue et al. (2022) in validating enzymatic assays against docking predictions [10]. Overcoming these issues requires improved algorithms, improved structural data, and integrated computational experimental workflows.

## 6. Future Directions and Perspectives

### 6.1. Innovations and Emerging Techniques

The future of PARP1 inhibitor design relies on the utilization of sophisticated computational techniques, including machine learning (ML) and artificial intelligence (AI). Gomatam et al. (2024) demonstrated that ML algorithms, such as Random Forests and Support Vector Machines, can take advantage of datasets comprising over 10,000 compounds to well predict PARP1 inhibitory activity at 95% [5]. Deep learning programs, for example, those of Shahab et al. (2025), employ convolutional neural networks to identify novel scaffolds with IC_50_ values of 5 nM or lower, expanding the chemical space of PARP1 inhibitors [6]. Generative AI programs, such as variational autoencoders and GANs, enable de novo inhibition design with optimal lipophilicity and selectivity, exemplified by Khizer et al. (2024) via piperidine-based inhibitors [4]. In addition, quantum computing will accelerate DFT and TD-DFT computations and, thus, reduce computation time for large biomolecular systems by 50% [23]. Integrate multi-omics analysis, which combines genomic, proteomic, and metabolomic data, may also enable patient-specific inhibitor design, as Lin et al. (2024) have proposed for personalized therapy of breast cancer [14]. These technologies, in conjunction with high-throughput virtual screening, will drive the discovery of next-generation PARP1 inhibitors with enhanced efficacy and reduced toxicity.

### 6.2. Integrating Computational and Experimental Approaches

Successful identification of PARP1 inhibitors relies on the cooperative harmonic interaction between predictive computational and experimental validation to convert in silico results into therapeutically potent treatments. Computational tools such as molecular docking, MD simulations, QSAR models and ML provide a great platform for the identification and optimization of selective and potent PARP1 inhibitors. For instance, Bhatnagar et al. (2023) used a sequential approach of e-pharmacophore modeling followed by molecular docking to virtually screen 1.2 million compounds and found a lead inhibitor with a docking score of −8.5 kcal/mol that was also validated by in vitro enzymatic assays with an IC_50_ value of 12 nM in *BRCA*-mutated breast cancer cells [8]. Similarly, Xue et al. (2022) also predicted a two-step PARP1-DNA trapping mechanism by docking, which was subsequently confirmed by in vitro assays that their inhibitor trapped PARP1 at DNA damage and suppressed cell viability by 70% in *BRCA1/2*-deficient cell lines [10]. These predictions allow for the selection of candidate molecules, reducing the experimental load significantly by prioritizing compounds to be synthesized and tested.

Experimental confirmation is needed to validate computational predictions and find out about biological activity, pharmacokinetics, and safety. Binding affinity and efficacy are quantified through in-vitro assays such as enzymatic inhibition and cell viability assays. For example, Khizer et al. (2024) utilized MD simulations to identify a piperidine-based inhibitor with stable hydrogen bonds to Gly863 and Ser904 (which persisted for 85% of a 100 ns simulation), and in vitro assays where an IC_50_ of 10 nM was established [4]. In vivo studies also assess therapeutic efficacy and toxicity, as demonstrated by Shahab et al. (2025), who validated a deep learning-designed inhibitor (IC_50_ = 5 nM) in xenograft models with 65% inhibition of tumor growth in *BRCA*-mutated breast cancer [6]. High-throughput screening platforms, such as fluorescence-based PARP1 activity assays, complement computational tools by experimentally screening many candidates rapidly, as exemplified by Lin et al. (2024), who experimentally confirmed piperidine derivatives with a 75% inhibition rate [14].

The interplay between experimental and computational approaches enhances inhibitor optimization. Computational optimizations, such as QSAR-guided structural refinements, direct the synthesis of analogs with improved properties. Gomatam et al. (2024) utilized ML-driven QSAR models (R^2^ = 0.95) to predict the impact of lipophilic substituents on the inhibition of PARP1, leading to the synthesis of a quinazolinone analog with a 20% increase in potency (IC_50_ = 8 nM) [5]. Conversely, experimental data refine computational models by identifying inconsistencies, e.g., off-target activities or solubility issues, as noted by Sadybekov and Katritch (2023) [15]. For instance, Hirlekar et al. (2023) revised their docking protocol after in vitro studies revealed 10% overestimation of binding affinity due to unmodeled solvent effects [1]. Emerging technologies, such as CRISPR-based functional genomics and single-cell RNA sequencing, further bridge the gap by validating inhibitor effects on DNA repair pathways in patient-derived cells, enabling personalized therapy design [24]. Patient-derived xenografts and organoids also augment this approach by providing physiologically relevant models for inhibitor efficacy validation, as shown by Lin et al. (2024) where they employed breast cancer organoids to confirm the 75% inhibition rate of piperidine-based inhibitors in *BRCA*-mutated cells [14]. This multi-pronged approach ensures that not only are the PARP1 inhibitors computationally optimized, but also experimentally viable, paving the way for precision oncology.

## 7. Conclusions

Incorporation of computational chemistry has revolutionized the drug development paradigm for PARP1 inhibitors, allowing for unmatched accuracy and efficacy in designing therapies for breast cancer, particularly in *BRCA1/2*-mutated tumors [25]. Techniques such as molecular docking, MD simulations, QSAR modeling, DFT/TD-DFT calculations, and ML-facilitated virtual screening have accelerated discovery and optimization of effective inhibitors, evidenced by approved drugs including Olaparib (IC_50_ = 5 nM), Rucaparib (IC_50_ = 7 nM), Niraparib (IC_50_ = 7 nM), and Talazoparib (IC_50_ = 1 nM). Such methods have enabled the identification of novel scaffolds, such as quinazolinones and piperidine analogs, with −9.5 kcal/mol binding free energy and up to 90% inhibition rates in *BRCA*-deficient cells [26,27]. The union of computational predictions with experimental validation has given science some exciting milestones, such as 60–80% inhibition of tumor growth in pre-clinical models and improved progression-free survival in the clinic. Despite these advances, challenges remain, including the computational cost of large-scale simulations and the need for good structural data to make sound predictions [28]. The interplay between experiments and computations has been the key to overcoming this, as seen in the validation of docking predictions using in vitro and in vivo essays. Additionally, the future of PARP1 inhibitor development bodes well for further innovation. Emerging technologies, such as AI-driven generative models, quantum computing, and multi-omics integration, can potentially enhance the development of inhibitors with greater selectivity, reduced toxicity, and resistance-reducing activity, including strategies to overcome PARP1-mediated resistance by allosteric inhibition and binding-optimized interactions, such as demonstrated for up to 30% improved selectivity in resistant cell lines. For instance, Shahab et al. (2025) used deep learning to design inhibitors with 97% prediction accuracy, opening new avenues for personalized therapy [6]. Utilization of patient-specific data, such as genomic and proteomic profiles, may make subsequent personalized inhibitor design feasible, paving the way for a new era in precision oncology. With further advancements in computational and experimental methods, synergy between these fields will drive the development of next-generation PARP1 inhibitors, promoting revolutionary targeted cancer treatments and improved prognosis for breast cancer patients worldwide.

## Figures and Tables

**Table 1:** Summary of Key PARP1 Inhibitors and References from Case Studies.

PARP1 Inhibitor	Description	Computational Methods	References
**Olaparib**	First FDA-approved PARP1 inhibitor for *BRCA*-mutated breast and ovarian cancers; IC_50_ = 5 nM.	Molecular docking, MD simulations, QSAR, virtual screening	[4,15]
**Rucaparib**	Approved for ovarian cancer; high selectivity for PARP1 (15:1 vs. PARP2); IC_50_ = 7 nM.	Virtual screening, MD simulations, docking	[1,8]
**Niraparib**	Approved for maintenance therapy in ovarian cancer; IC_50_ = 7 nM; 65% reduction in cell viability.	Molecular docking, 3D-QSAR, MD simulations	[8]
**Talazoparib**	Highly potent inhibitor for *BRCA*-mutated breast cancer; IC_50_ = 1 nM; 62% objective response rate.	Deep learning, docking, MD simulations, TD-DFT	[6]
**Quinazolinone Analogs**	Novel inhibitors with IC_50_ = 10 nM; promising for *BRCA*-mutated breast cancer.	TD-DFT, molecular docking, MD simulations	[9]
**Piperidine Derivatives**	Novel scaffold with IC_50_ = 12 nM; 75% inhibition in vitro.	De Novo design, MD simulations, docking	[14]
**Olaparib–Chlorambucil**	Hybrid inhibitor with synergistic effects; 90% reduction in cell viability in *BRCA*-deficient cells.	DFT, molecular docking	[17]

**Table 2:** Comparative Structural highlights.

Drug/Scaffold	Core Scaffold	Key Functional Groups	Lipophilicity (LogP)	Binding Strength	Unique Feature
**Olaparib**	Phthalazinone	Carbonyl, piperazine, fluorophenyl	~2.5	High	Balanced π-stacking + H-bonds
**Rucaparib**	tricyclic indole-fused lactam	Amide, chlorophenyl, piperazine	~2.7	High	Stronger aromatic contacts
**Niraparib**	2H-indazole-7-carboxamide scaffold	Trifluoromethyl, piperidine, amide	~3.1	High	Hydrophobic boosting potency
**Talazoparib**	Tricyclic pyridazinone scaffold	Triazole, lactam, fluorophenyl	~2.4	Very High	Exceptional PARP trapping
**Quinazolinones**	Benzopyrimidinone scaffold	Carbonyl, amide, aromatic ring	~2.3–2.8	Medium–High	NAD^+^ mimetics for alternative scaffold
**Piperidine derivatives**	Piperidine hybrids	Flexible amine, aromatic linkers	Variable	Adjustable	Improves solubility & PK
**Olaparib–Chlorambucil**	Hybrid scaffold	Nitrogen mustard + phthalazinone	~3.0	Very High	Dual DNA damage + PARP inhibition

## Data Availability

No new data were created or analyzed in this study. Data sharing is not applicable to this article.

## Abbreviations

The following abbreviations are used in this manuscript:

PARP1	Poly (ADP-ribose) polymerase 1
DNA	Deoxyribonucleic Acid
RNA	Ribonucleic Acid
NAD^+^	Nicotinamide adenine dinucleotide
*BRCA*	Breast Cancer Gene
HRR	Homologous Recombination Repair
QSAR	Quantitative Structure Activity Relationship
BER	Base Excision Repair
MD	Molecular Dynamics
DFT	Density Functional Theory
TD-DFT	Time Dependent–Density Functional Theory
SSB	Single-strand Break
DSB	Double-strand Break
GPU	Graphical Processing Unit
HD	Helical domain
